# Crystal structure of *trans*-dihydrido­bis[tris­(di­methyl­amino)­phosphane-κ*P*]platinum(II)

**DOI:** 10.1107/S2056989015004351

**Published:** 2015-03-14

**Authors:** Emma L. Downs, Lev N. Zakharov, David R. Tyler

**Affiliations:** a Department of Chemistry and Biochemistry, 1253 University of Oregon, Eugene, Oregon 97403-1253, USA

**Keywords:** crystal structure, tris­(di­methyl­amino)­phosphane, platinum(II) complex, ligand-assisted hydration, nitrile hydration

## Abstract

The mol­ecule of the title compound, [PtH_2_(C_6_H_18_N_3_P)_2_], has a centrosymmetric square-planar structure in which the Pt^II^ atom is bonded to two H and two P atoms in a mutually *trans* configuration. The Pt^II^ atom sits on an inversion center and thus the asymmetric unit contains only half the mol­ecule. The Pt—P and Pt—H distances are 2.2574 (10) and 1.49 (7) Å, respectively.

## Related literature   

For the synthesis of related compounds, see: Packett *et al.* (1985 ▸). For information on ligand-assisted hydration, see: Grotjahn (2005 ▸); Grotjahn *et al.* (2008*a* ▸,*b* ▸). For further information on nitrile hydration, see: García-Álvarez *et al.* (2011 ▸); Knapp *et al.* (2012 ▸, 2013*a* ▸,*b* ▸). For a review of the literature on nitrile hydration, see: Ahmed *et al.* (2011 ▸). For related structures, see: Packett *et al.* (1985 ▸); Robertson *et al.* (1986 ▸); Ferguson *et al.* (1979 ▸).
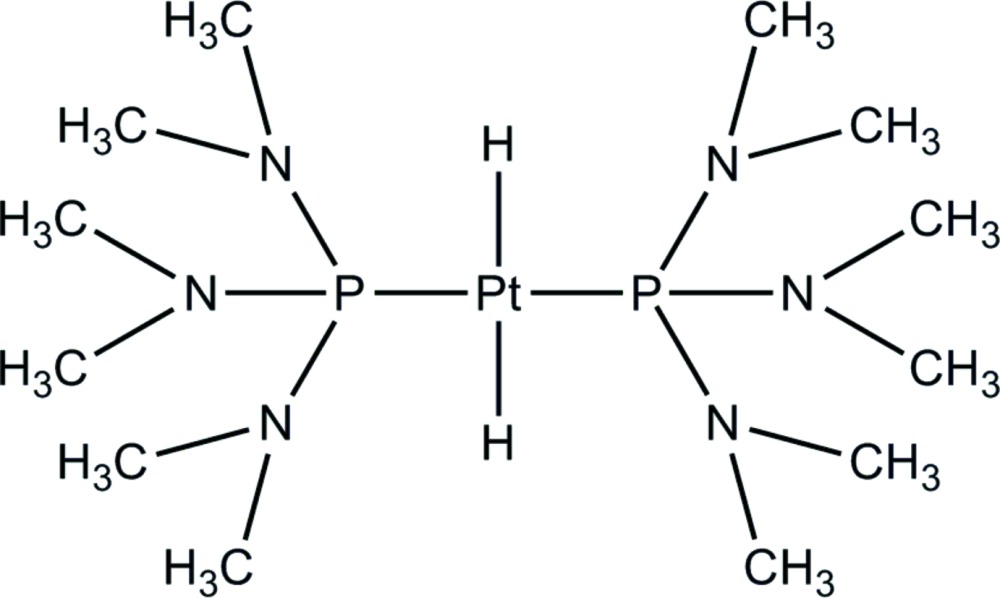


## Experimental   

### Crystal data  

[PtH_2_(C_6_H_18_N_3_P)_2_]
*M* *_r_* = 523.51
Triclinic, 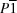

*a* = 7.8871 (19) Å
*b* = 7.9499 (19) Å
*c* = 9.891 (2) Å
α = 76.807 (4)°
β = 73.241 (4)°
γ = 60.652 (3)°
*V* = 514.8 (2) Å^3^
*Z* = 1
Mo *K*α radiation
μ = 6.97 mm^−1^
*T* = 173 K
0.08 × 0.06 × 0.03 mm

### Data collection  

Bruker APEXII CCD area-detector diffractometer
Absorption correction: multi-scan (*SADABS*; Sheldrick, 1995 ▸) *T*_min_ = 0.856, *T*_max_ = 1.000
5813 measured reflections
2238 independent reflections
2238 reflections with *I* > 2σ(*I*)
*R*_int_ = 0.020

### Refinement  

*R*[*F* ^2^ > 2σ(*F* ^2^)] = 0.023
*wR*(*F* ^2^) = 0.059
*S* = 1.04
2238 reflections
101 parameters
H atoms treated by a mixture of independent and constrained refinement
Δρ_max_ = 0.65 e Å^−3^
Δρ_min_ = −0.69 e Å^−3^

### 

Data collection: *APEX2* (Bruker, 2008 ▸); cell refinement: *SAINT* (Bruker, 2000 ▸); data reduction: *SAINT*; program(s) used to solve structure: *SHELXTL* (Sheldrick, 2008 ▸); program(s) used to refine structure: *SHELXTL*; molecular graphics: *SHELXTL*; software used to prepare material for publication: *SHELXTL*.

## Supplementary Material

Crystal structure: contains datablock(s) I. DOI: 10.1107/S2056989015004351/pk2545sup1.cif

**Fig. 1.**
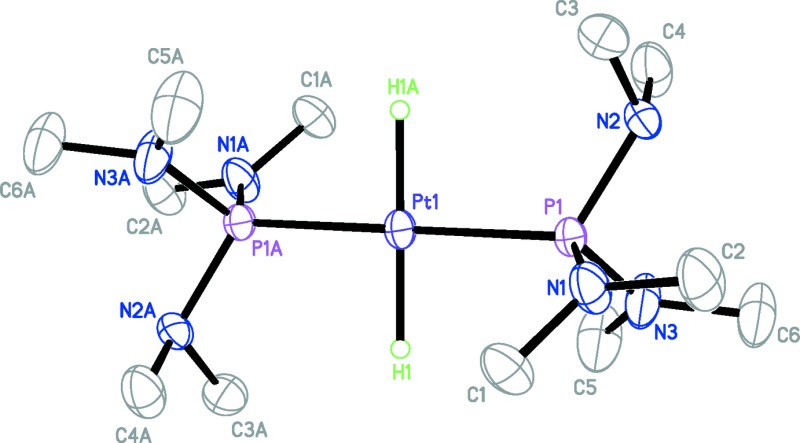
The crystal structure of trans-dihydridobis[tris(dimethylamino)phosphane]platinum (II) with 50% probability displacement ellipsoids. H atoms in the Me groups are omitted for clarity. [Symmetry code (A): 1 - x, 2 - y, 1 - z].

Structure factors: contains datablock(s) I. DOI: 10.1107/S2056989015004351/pk2545Isup2.hkl

trans x y z . DOI: 10.1107/S2056989015004351/pk2545fig1.tif
The crystal structure of *trans*-dihydridobis[tris­(di­methyl­amino)­phosphane]platinum (II) with 50% probability displacement ellipsoids. H atoms in the Me groups are omitted for clarity. [Symmetry code (A): 1 − *x*, 2 − *y*, 1 − *z*].

CCDC reference: 1051841

Additional supporting information:  crystallographic information; 3D view; checkCIF report

## supplementary crystallographic information

### S1. Comment

The hydration of nitriles using homogeneous catalysts is often too slow for practical applications (Ahmed *et al.*, 2011). Hydroxide is a much better nucleophile than water, and thus to increase the rate, many hydration reactions are carried out at high *p*H. When a ligand on the catalyst is capable of hydrogen bonding, the entering water nucleophile can be activated by hydrogen bonding interactions, avoiding the need for strongly basic solutions. Large rate accelerations in hydration reactions have been observed and attributed to this phenomenon, known as ligand assisted hydration or bifunctional catalysis (Grotjahn, 2005; Grotjahn *et al.*, 2008*a*,b). Complexes with phosphane ligands containing hydrogen bonding moieties, in particular tris(dimethylamino)phosphane (P(NMe_2_)_3_), have achieved excellent results in nitrile hydration reactions (García-Álvarez *et al.*, 2011; Knapp *et al.*, 2012, 2013*a*,b). In particular, we reported that the [RuCl_2_(η^6^-*p*-cymene){P(NMe_2_)_3_}] complex is an excellent nitrile hydration catalyst (Knapp *et al.*, 2012). Unlike related catalysts, this complex was active under acidic conditions (*p*H 3.5), and the improved stability of cyanohydrins in an acidic medium yielded excellent results. Glycolonitrile (1) and lactonitrile (2) were hydrated fully to their corresponding amides and acetone cyanohydrin (3) was converted to 3-hydroxy-isobutyro nitrile (HIBAM) in 15% yield. Based on this result, we hypothesized that the tris(dimethylamino)phosphane ligand could be used in other homogeneous catalysts to enhance the rates of hydration. For this purpose, two new platinum complexes, Pt(H)(Cl)(P(NMe_2_)_3_)_2_ and Pt(H)_2_(P(NMe_2_)_3_)_2_, were synthesized and tested for hydration activity with a variety of nitriles, including aromatic and aliphatic nitriles and cyanohydrins.

Pt(H)_2_(P(NMe_2_)_3_)_2_ was characterized by single-crystal X-ray diffraction methods. The molecule has a square planar structure (P(1)(1 - *x*,2 - *y*,1 - *z*)-Pt(1) (*x*,*y*,*z*) –P(1)(*x*,*y*,*z*) = 180.0 °). The Pt—P bond lengths (2.2572 (8) Å) are comparable to other Pt(H)_2_(phosphane)_2_ complexes: Pt(H)_2_(PMe_3_)_2_, 2.259 (3) Å; Pt(P*i*Pr_3_)_2_(H)_2_, 2.252 (1) Å; Pt(H)_2_(P*t*Bu_3_)_2_, 2.276 (3) Å. (Packett *et al.*, 1985; Robertson *et al.*, 1986; Ferguson *et al.*, 1979). The P atom coordination environments are slightly distorted tetrahedral: N(3)—P(1)—N(1) = 110.86 (15)°; N(3)—P(1)—N(2) = 100.94 (14)°; N(1)—P(1)—N(2) = 98.70 (13)°; N(3)—P(1)—Pt(1) = 112.12 (10)°; N(1)—P(1)—Pt(1) = 113.82 (9)°; N(2)—P(1)—Pt(1) = 119.08 (9)°). The three NMe_2_ groups bonded to each P atom have a staggered orientation with respect to the three NMe_2_ groups on the other P atom. Consequently, the two Pt—P—N(2) angles, with atoms in the same plane as the Pt—H bonds, are significantly distorted (119.08 (9)°) from the tetrahedral angle.

### S2. Experimental

Synthesis of Pt(H)_2_(P(NMe_2_)_3_)_2_. In an inert atmosphere, PtCl_2_(COD) (0.1 g, 0.27 mmol) was dissolved in 10 ml dichloromethane. Two equivalents of P(NMe_2_)_3_ (0.1 ml, 0.54 mmol) were added dropwise with stirring. The solution turned from colorless to light yellow. The solution was stirred overnight. ^31^P NMR confirmed the formation of *cis*-PtCl_2_(P(NMe_2_)_3_)_2_: the free phosphane peak at 122 p.p.m. had disappeared and a peak with platinum satellites at 60 p.p.m. had appeared. The solvent and COD were removed *in vacuo* and the resulting light yellow powder was redissolved in acetonitrile. Two equivalents (0.02 g, 0.54 mmol) of NaBH_4_ were added with stirring. The solution was stirred for two hours and became bright orange; solids began to precipitate. The mixture was filtered through a celite plug to remove solids, and the solvent was removed. The brown solid was redissolved in minimal acetone and layered on top of water to precipitate brown crystals. ^31^P NMR: 129 p.p.m., Pt satellites at 138, 120 p.p.m.. J_Pt—P_ = 1,891 Hz. ^1^H NMR: t, 2.8 p.p.m. (J_P—H_ = 5.5 Hz), tt, -3.5 (J_P—H_ = 17.5 Hz, J_Pt—H_ = 405 Hz).

### S3. Refinement

The structure was solved using direct methods and refined with anisotropic thermal parameters for non-H atoms. The H atom bonded to the Pt atom was found in the residual density and refined with isotropic thermal parameters. H atoms in the Me groups were positioned geometrically and refined using a rigid group model: C—H = 0.98 Å, *U*_iso_(H) = 1.5*U*_eq_(C).

### Crystal data

**Table:**

[PtH_2_(C_6_H_18_N_3_P)_2_]	*Z* = 1
*M**_r_* = 523.51	*F*(000) = 260
Triclinic, *P*1	*D*_x_ = 1.689 Mg m^−^^3^
*a* = 7.8871 (19) Å	Mo *K*α radiation, λ = 0.71073 Å
*b* = 7.9499 (19) Å	Cell parameters from 3285 reflections
*c* = 9.891 (2) Å	θ = 3.0–26.9°
α = 76.807 (4)°	µ = 6.97 mm^−^^1^
β = 73.241 (4)°	*T* = 173 K
γ = 60.652 (3)°	Block, colorless
*V* = 514.8 (2) Å^3^	0.08 × 0.06 × 0.03 mm

### Data collection

**Table:**

Bruker APEXII CCD area-detector diffractometer	2238 reflections with *I* > 2σ(*I*)
Radiation source: Sealed tube with triumph monochromator	*R*_int_ = 0.020
φ and ω scans	θ_max_ = 27.0°, θ_min_ = 2.2°
Absorption correction: multi-scan (*SADABS*; Sheldrick, 1995)	*h* = −10→10
*T*_min_ = 0.856, *T*_max_ = 1.000	*k* = −10→10
5813 measured reflections	*l* = −12→12
2238 independent reflections	

### Refinement

**Table:**

Refinement on *F*^2^	0 restraints
Least-squares matrix: full	Hydrogen site location: mixed
*R*[*F*^2^ > 2σ(*F*^2^)] = 0.023	H atoms treated by a mixture of independent and constrained refinement
*wR*(*F*^2^) = 0.059	*w* = 1/[σ^2^(*F*_o_^2^) + (0.0425*P*)^2^] where *P* = (*F*_o_^2^ + 2*F*_c_^2^)/3
*S* = 1.04	(Δ/σ)_max_ < 0.001
2238 reflections	Δρ_max_ = 0.65 e Å^−^^3^
101 parameters	Δρ_min_ = −0.69 e Å^−^^3^

### Special details

**Table:**

Geometry. All e.s.d.'s (except the e.s.d. in the dihedral angle between two l.s. planes) are estimated using the full covariance matrix. The cell e.s.d.'s are taken into account individually in the estimation of e.s.d.'s in distances, angles and torsion angles; correlations between e.s.d.'s in cell parameters are only used when they are defined by crystal symmetry. An approximate (isotropic) treatment of cell e.s.d.'s is used for estimating e.s.d.'s involving l.s. planes.

### Fractional atomic coordinates and isotropic or equivalent isotropic displacement parameters (Å^2^)

**Table:**

	*x*	*y*	*z*	*U*_iso_*/*U*_eq_	
Pt1	0.5000	1.0000	0.5000	0.02501 (8)	
P1	0.58089 (14)	0.80751 (13)	0.70124 (10)	0.02302 (18)	
N1	0.8017 (5)	0.7594 (5)	0.7244 (4)	0.0332 (7)	
N2	0.6130 (5)	0.5757 (4)	0.7200 (3)	0.0272 (6)	
N3	0.4051 (6)	0.8932 (5)	0.8436 (4)	0.0405 (9)	
C1	0.8862 (7)	0.8928 (6)	0.6632 (5)	0.0372 (9)	
H1A	1.0160	0.8404	0.6880	0.056*	
H1B	0.7971	1.0185	0.7007	0.056*	
H1C	0.9028	0.9096	0.5597	0.056*	
C2	0.9106 (7)	0.6069 (7)	0.8243 (5)	0.0433 (11)	
H2A	1.0362	0.6085	0.8177	0.065*	
H2B	0.9382	0.4808	0.8019	0.065*	
H2C	0.8308	0.6284	0.9209	0.065*	
C3	0.7765 (7)	0.4524 (6)	0.6164 (5)	0.0400 (10)	
H3A	0.7849	0.3224	0.6347	0.060*	
H3B	0.9016	0.4438	0.6244	0.060*	
H3C	0.7529	0.5083	0.5205	0.060*	
C4	0.4314 (7)	0.5651 (7)	0.7249 (5)	0.0433 (11)	
H4A	0.4592	0.4288	0.7360	0.065*	
H4B	0.3844	0.6298	0.6366	0.065*	
H4C	0.3289	0.6295	0.8055	0.065*	
C5	0.2224 (7)	1.0666 (8)	0.8357 (6)	0.0543 (14)	
H5B	0.1401	1.0908	0.9314	0.081*	
H5C	0.1509	1.0512	0.7769	0.081*	
H5D	0.2511	1.1764	0.7935	0.081*	
C6	0.4176 (8)	0.7934 (7)	0.9864 (5)	0.0488 (12)	
H6C	0.2983	0.8692	1.0539	0.073*	
H6D	0.5356	0.7789	1.0119	0.073*	
H6A	0.4274	0.6649	0.9892	0.073*	
H1	0.490 (10)	1.163 (10)	0.557 (7)	0.070 (19)*	

### Atomic displacement parameters (Å^2^)

**Table:**

	*U*^11^	*U*^22^	*U*^33^	*U*^12^	*U*^13^	*U*^23^
Pt1	0.03283 (12)	0.02060 (11)	0.02016 (11)	−0.01088 (8)	−0.01106 (8)	0.00457 (7)
P1	0.0288 (4)	0.0194 (4)	0.0196 (4)	−0.0097 (4)	−0.0092 (3)	0.0027 (3)
N1	0.0396 (18)	0.0315 (17)	0.0374 (19)	−0.0219 (15)	−0.0225 (15)	0.0127 (14)
N2	0.0358 (17)	0.0198 (15)	0.0279 (16)	−0.0138 (13)	−0.0115 (13)	0.0031 (12)
N3	0.043 (2)	0.0329 (19)	0.0202 (16)	−0.0017 (16)	−0.0033 (14)	0.0009 (14)
C1	0.037 (2)	0.034 (2)	0.047 (2)	−0.0233 (18)	−0.0090 (18)	0.0020 (18)
C2	0.047 (3)	0.042 (2)	0.048 (3)	−0.024 (2)	−0.030 (2)	0.017 (2)
C3	0.050 (3)	0.024 (2)	0.039 (2)	−0.0111 (18)	−0.0111 (19)	−0.0037 (17)
C4	0.052 (3)	0.050 (3)	0.042 (2)	−0.035 (2)	−0.023 (2)	0.014 (2)
C5	0.040 (2)	0.047 (3)	0.041 (3)	0.003 (2)	−0.003 (2)	−0.001 (2)
C6	0.054 (3)	0.043 (3)	0.024 (2)	−0.008 (2)	−0.0055 (19)	0.0046 (18)

### Geometric parameters (Å, º)

**Table:**

Pt1—P1	2.2574 (10)	C2—H2A	0.9800
Pt1—P1^i^	2.2574 (10)	C2—H2B	0.9800
Pt1—H1	1.49 (7)	C2—H2C	0.9800
P1—N3	1.660 (4)	C3—H3A	0.9800
P1—N1	1.664 (3)	C3—H3B	0.9800
P1—N2	1.705 (3)	C3—H3C	0.9800
N1—C1	1.450 (5)	C4—H4A	0.9800
N1—C2	1.451 (5)	C4—H4B	0.9800
N2—C3	1.460 (5)	C4—H4C	0.9800
N2—C4	1.462 (5)	C5—H5B	0.9800
N3—C5	1.432 (6)	C5—H5C	0.9800
N3—C6	1.458 (6)	C5—H5D	0.9800
C1—H1A	0.9800	C6—H6C	0.9800
C1—H1B	0.9800	C6—H6D	0.9800
C1—H1C	0.9800	C6—H6A	0.9800
			
P1—Pt1—P1^i^	180.0	N1—C2—H2C	109.5
P1—Pt1—H1	90 (3)	H2A—C2—H2C	109.5
P1^i^—Pt1—H1	90 (3)	H2B—C2—H2C	109.5
N3—P1—N1	110.9 (2)	N2—C3—H3A	109.5
N3—P1—N2	101.05 (19)	N2—C3—H3B	109.5
N1—P1—N2	98.82 (17)	H3A—C3—H3B	109.5
N3—P1—Pt1	112.10 (13)	N2—C3—H3C	109.5
N1—P1—Pt1	113.77 (12)	H3A—C3—H3C	109.5
N2—P1—Pt1	118.93 (12)	H3B—C3—H3C	109.5
C1—N1—C2	112.8 (3)	N2—C4—H4A	109.5
C1—N1—P1	121.1 (3)	N2—C4—H4B	109.5
C2—N1—P1	125.2 (3)	H4A—C4—H4B	109.5
C3—N2—C4	110.0 (4)	N2—C4—H4C	109.5
C3—N2—P1	114.7 (3)	H4A—C4—H4C	109.5
C4—N2—P1	113.4 (3)	H4B—C4—H4C	109.5
C5—N3—C6	114.0 (4)	N3—C5—H5B	109.5
C5—N3—P1	122.7 (3)	N3—C5—H5C	109.5
C6—N3—P1	123.1 (3)	H5B—C5—H5C	109.5
N1—C1—H1A	109.5	N3—C5—H5D	109.5
N1—C1—H1B	109.5	H5B—C5—H5D	109.5
H1A—C1—H1B	109.5	H5C—C5—H5D	109.5
N1—C1—H1C	109.5	N3—C6—H6C	109.5
H1A—C1—H1C	109.5	N3—C6—H6D	109.5
H1B—C1—H1C	109.5	H6C—C6—H6D	109.5
N1—C2—H2A	109.5	N3—C6—H6A	109.5
N1—C2—H2B	109.5	H6C—C6—H6A	109.5
H2A—C2—H2B	109.5	H6D—C6—H6A	109.5
			
N3—P1—N1—C1	−100.5 (4)	N3—P1—N2—C4	58.6 (3)
N2—P1—N1—C1	154.0 (3)	N1—P1—N2—C4	172.0 (3)
Pt1—P1—N1—C1	26.9 (4)	Pt1—P1—N2—C4	−64.5 (3)
N3—P1—N1—C2	67.7 (4)	N1—P1—N3—C5	130.0 (4)
N2—P1—N1—C2	−37.8 (4)	N2—P1—N3—C5	−126.0 (5)
Pt1—P1—N1—C2	−164.9 (3)	Pt1—P1—N3—C5	1.7 (5)
N3—P1—N2—C3	−174.0 (3)	N1—P1—N3—C6	−53.6 (5)
N1—P1—N2—C3	−60.5 (3)	N2—P1—N3—C6	50.4 (5)
Pt1—P1—N2—C3	62.9 (3)	Pt1—P1—N3—C6	178.1 (4)

Symmetry code: (i) −*x*+1, −*y*+2, −*z*+1.
