# Supplementary figures and images for: Crystal structure of trans-dihydrido­bis[tris­(di­methyl­amino)­phosphane-κP]platinum(II)
Source: Acta Crystallogr E Crystallogr Commun. 2015 Mar 14;71(Pt 4):m83–4. doi: 10.1107/S2056989015004351 (PMC4438795; doi:10.1107/S2056989015004351)

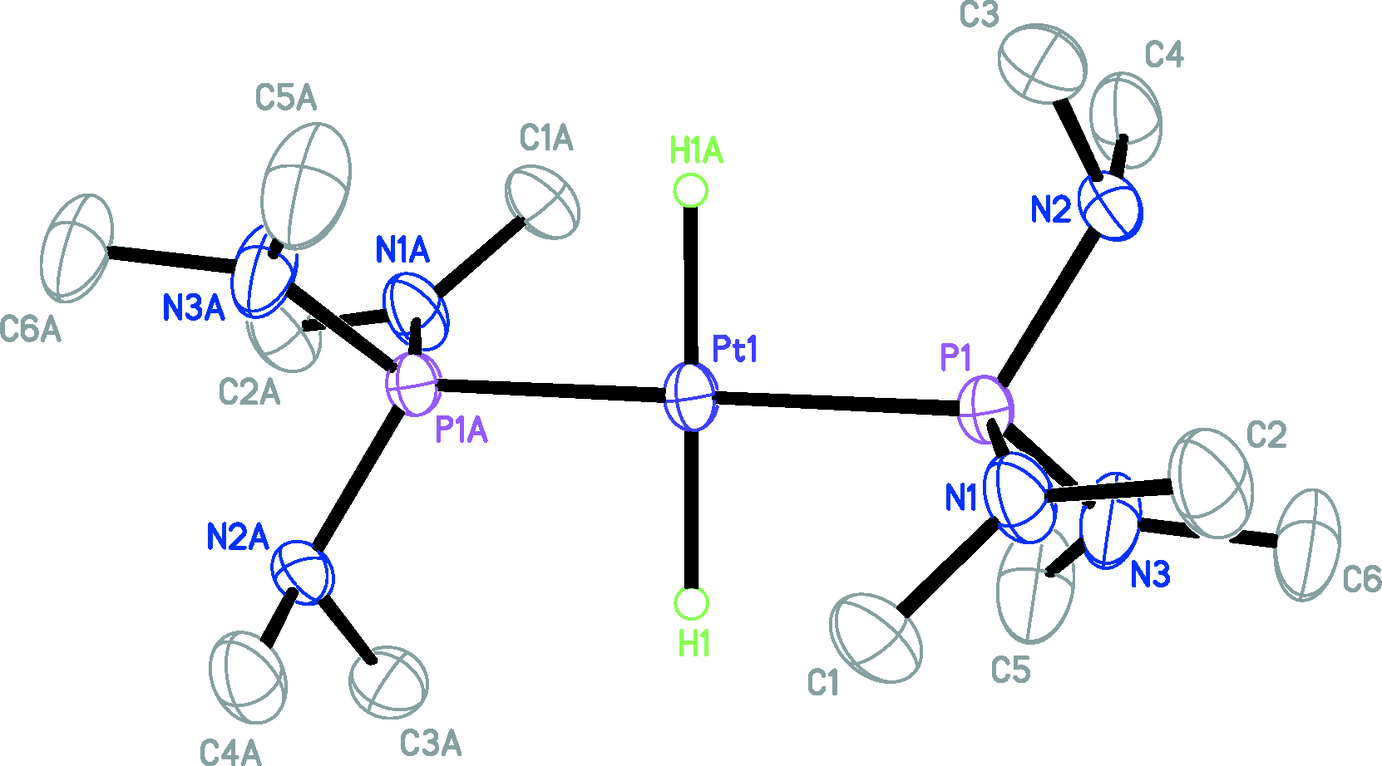

Supplement: Supplementary file 3 [file e-71-00m83-fig1.tif]
